# Luminescent lanthanide metallopeptides for biomolecule sensing and cellular imaging

**DOI:** 10.1039/d4cc03205e

**Published:** 2024-09-27

**Authors:** Rosalía Sánchez-Fernández, Ines Obregon-Gomez, Axel Sarmiento, M. Eugenio Vázquez, Elena Pazos

**Affiliations:** a CICA – Centro Interdisciplinar de Química e Bioloxía and Departamento de Química, Facultade de Ciencias, Universidade da Coruña 15071 A Coruña Spain elena.pazos@udc.es; b Centro Singular de Investigación en Química Biolóxica e Materiais Moleculares (CiQUS), Departamento de Química Orgánica, Universidade de Santiago de Compostela 15782 Santiago de Compostela Spain

## Abstract

Lanthanide ions display unique luminescent properties that make them particularly attractive for the development of bioprobes, including long-lived excited states that allow the implementation of time-gated experiments and the elimination of background fluorescence associated with biological media, as well as narrow emission bands in comparison with typical organic fluorophores, which allow ratiometric and multiplex assays. These luminescent complexes can be combined with peptide ligands to endow them with additional targeting, responsiveness, and selectivity, thus multiplying the opportunities for creative probe design. In this feature article we will present some of the main strategies that researchers have used to develop lanthanide metallopeptide probes for the detection of proteins and nucleic acids, as well as for monitoring enzymatic activity and cellular imaging.

## Abstract 2

Lanthanide ions display unique luminescent properties, such as long-lived excited states and relatively narrow emission bands, that make them particularly attractive for the development of bioprobes in combination with peptides.

## Graphical abstract

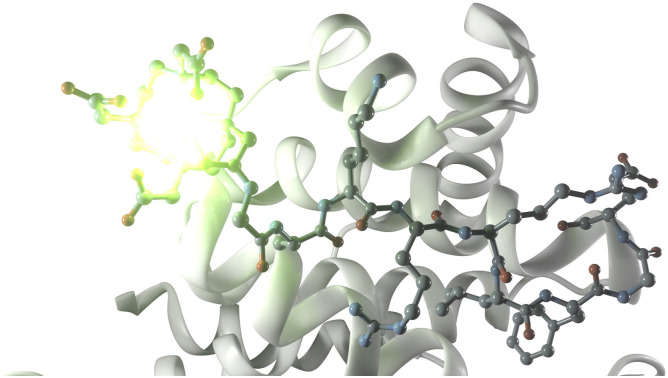

## Introduction

Over the last 30 years (photo)luminescent techniques have revolutionized scientific research, especially in the biochemical and biomedical fields. This success relies on their high sensitivity, short response times, and experimental simplicity. Furthermore, the specificity of the luminescent properties of the emitting species as a function of their microenvironment and their ability to provide spatial and temporal information, make these techniques particularly suited for biological or biomedical applications.^1^

The term luminescence refers to the spontaneous emission of radiation from an electronically excited species or from a vibrationally excited species not in thermal equilibrium with its environment.^2^ Different types of luminophores have been used to develop biosensors and imaging tools, including organic compounds^3–6^ and metal complexes, namely transition metal and lanthanide ion complexes.^7–13^ Unlike organic luminophores, lanthanide ion complexes have long lifetimes, ranging from microseconds to even milliseconds. Therefore, lanthanide complexes are very interesting luminescent species for biosensing and bioimaging, thanks to the possibility of eliminating the characteristic autofluorescence signal of biological samples by time-resolved luminescence.^8^ However, even though lanthanide complexes are highly versatile and can be endowed with different photophysical properties by modifying the metal ion or the ligands, it is often difficult to predict important parameters such as cell internalization, intracellular organelle distribution or affinity towards particular biomolecules, which must be considered for the rational design of biosensing and bioimaging tools. Peptides are essential biomolecules and, thanks to their synthetic simplicity, molecular recognition capabilities, and intrinsic biocompatibility, have emerged as excellent platforms for the preparation of drugs,^14–16^ biosensors,^17–20^ and imaging agents,^18,21,22^ among other applications. Therefore, their conjugation to metal complexes has emerged as an effective approach to enhance targeted cell internalization, organelle localization, and biomolecular recognition of such complexes.

In this feature article, we summarize the advances in the development of luminescent lanthanide metallopeptides for biomolecule sensing and cellular imaging. Although it is easy to find reviews in the literature describing the biological or biomedical applications of luminescent lanthanide complexes,^8,10,11,13,23–27^ these usually describe large families of lanthanide complexes modified with different organic scaffolds, such as small molecules, dendrimers, oligonucleotides, or peptides. There are only a few reviews dedicated exclusively to lanthanide metallopeptides, focusing mainly on their design^28,29^ or on their use to monitor kinase and phosphatase activity.^20^ Given the broad topic that we aim to cover, we will summarize the main photophysical properties of luminescent lanthanide complexes and analyze how these properties can be exploited to define specific strategies for obtaining biomolecule sensors. Lastly, we will also describe the application of some representative examples of lanthanide metallopeptides used for biomolecule sensing and cellular imaging.

## Strategies for the design of luminescent lanthanide metallopeptide-based biosensors

### Spectroscopic properties of luminescent lanthanide complexes

**Fig. 1:**
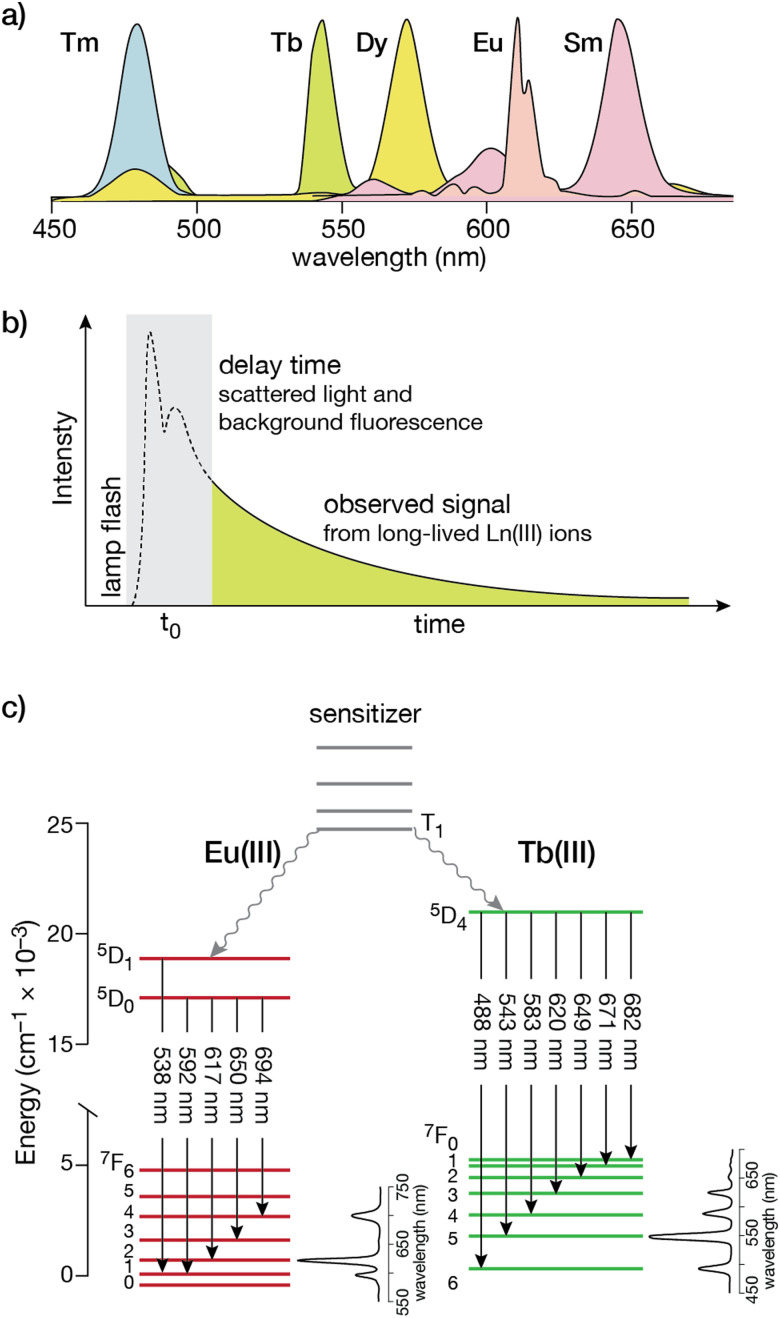
(a) Simplified photoluminescence spectra of UV/visible-emitting Ln(iii) ions. Fine structure in the emission bands is omitted for clarity; (b) a time-gated experiment that resolves the long-lived Ln(iii) luminescence from the autofluorescence of organic fluorophores in the sample; (c) electronic energy levels for Eu(iii) and Tb(iii). The transitions responsible for the different emission bands from the ^5^D states to the ground ^7^F manifold are indicated by the straight arrows, with typical intensity profiles of the emission bands shown to the right for each lanthanide. The non-radiative processes that couple the T_1_ state from the organic sensitizer to the excited states are shown as wavy arrows. Adapted from ref. 36 with permission from the Royal Society of Chemistry.

Lanthanide ion complexes are attractive luminescent species, but several aspects need to be considered for the successful design of luminescent lanthanide-metallopeptide biosensors. The unique spectroscopic properties of lanthanide ion complexes are due to transitions between 4f orbitals, which are shielded from the influence of the environment by the higher energy 5p and 6s orbitals, and are thus not involved in chemical bonding with the ligands.^13,30^ Compared with organic and transition metal complex luminophores, the isolation of the 4f orbitals results in narrower emission bands (with bandwidths of 15–30 nm), thereby facilitating multiplex experiments that simultaneously monitor more than one spectroscopic signal (Fig. 1a).^20,30,31^ Furthermore, these f–f transitions are forbidden by parity selection rules, so that the lifetimes of the lanthanide ions are very long, in the millisecond range in the case of Tb(iii) and Eu(iii), which is optimal for time-resolved experiments (Fig. 1b). However, for the same reason, lanthanide ions display very weak absorption bands, with molar absorption coefficients smaller than 15 M^−1^ × cm^−1^ (and generally below 3.5 M^−1^ × cm^−1^),^32^ making their direct excitation quite challenging.^13,20,33^ This limitation can be circumvented by using an indirect excitation mechanism, commonly known as “sensitization” or “antenna effect”. The sensitization process, which is particularly complex, involves the excitation of an organic chromophore followed by energy transfer through various pathways to one or more excited states of the lanthanide ion,^34^ resulting in a luminescent species.^33^ As simplified in Fig. 1c, it commonly involves several energy levels of the organic chromophore (frequently the lowest triplet state, T_1_, reached by intersystem crossing from the S_1_ excited state) and the lanthanide ion. Energy transfer may occur through two main processes: (i) Dexter's (or exchange) mechanism, which requires a good overlap between the orbitals of the chromophore and the lanthanide ion to accomplish a double electron transfer, or (ii) Förster's (or dipole–dipole) mechanism, in which the dipole moment of the T_1_ state of the chromophore couples with the dipole moment of the 4f orbital of the lanthanide ion, the latter being more likely for these metal ions.^20,35^ Based on this, most lanthanide-based biosensors and imaging agents are composed of the luminescent lanthanide ion, a coordinating ligand, and the sensitizing antenna.

### Fulfilling the requirements for lanthanide ion coordination with peptide ligands

**Fig. 2:**
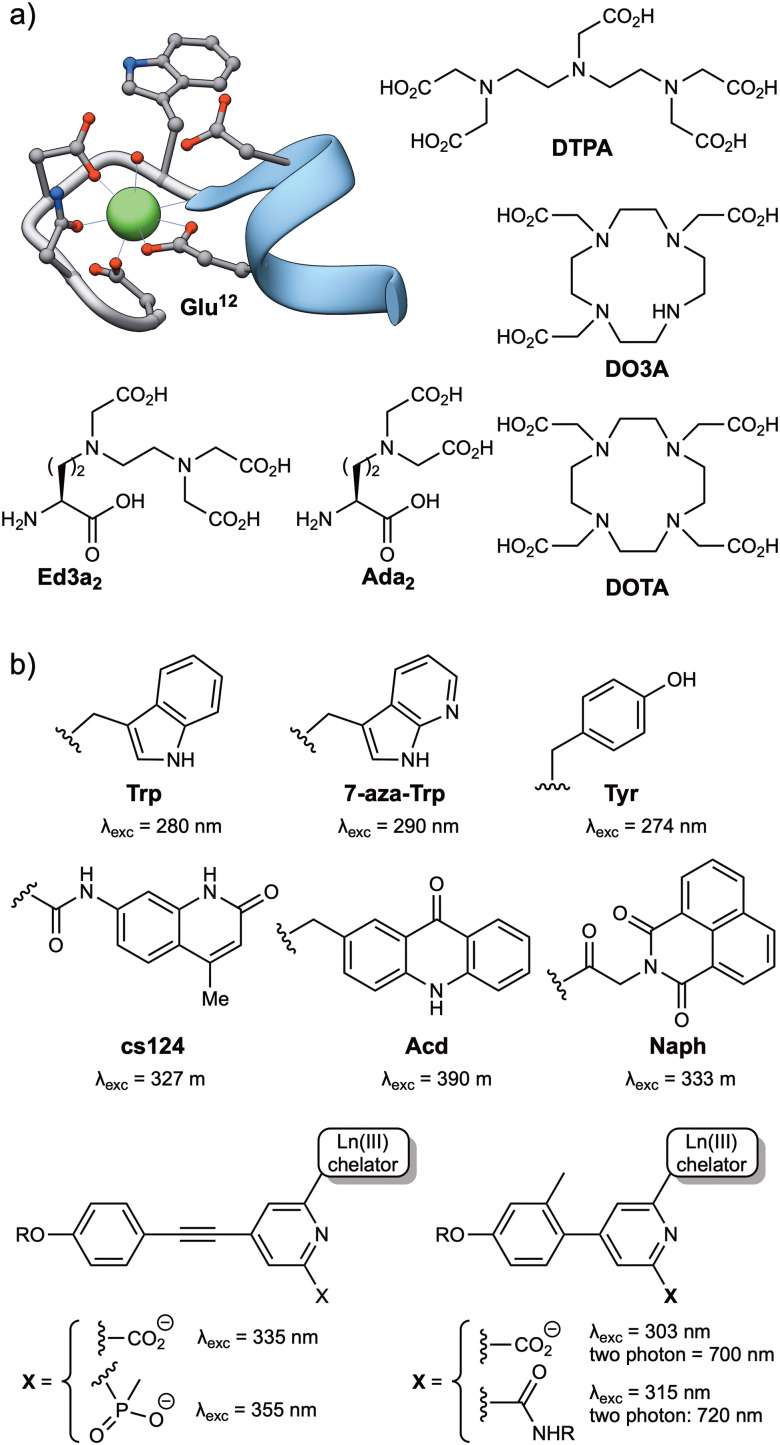
(a) Structures of commonly used Ln(iii) ligands, including EF-hand based peptides (residues 20 to 33 from the calmodulin structure, PDB: 1CLL) and polyaminocarboxylates (DTPA, DO3A, DOTA, Ed3a_2_ and Ada_2_); (b) typical Ln(iii) antennas, including naturally occurring amino acids (Trp, Tyr) and synthetic aromatic systems with appropriate energy levels to transfer their energy to the Ln(iii) ions (7-aza-Trp, cs124, Acd, Naph and the aryl–alkynyl–pyridine or aryl–pyridine derivatives).

Among the lanthanide ions, Tb(iii) and Eu(iii) are the most commonly used for the development of biosensors due to their long lifetimes, higher emission intensities, and lower sensitivity to quenching by vibrational energy transfer to X–H (X = C, N, O) and by singlet oxygen.^26^ Like all lanthanide ions, Tb(iii) and Eu(iii) have high coordination numbers (generally 8 or 9),^37^ and behave as hard Lewis acids, so the peptide ligands must incorporate a sufficient number of hard donor atoms, such as oxygen, to satisfy their coordination requirements. Tb(iii) is effectively sensitized by the phenol and indole side chains of tyrosine (Tyr) and tryptophan (Trp), respectively, and these natural amino acids have been widely used as antennas for the development of Tb(iii)-metallopeptide biosensors. Additionally, extrinsic sensitizers, such as 7-azatryptophan,^38^ 1,8-naphthalimide^39^ or carbostyril 124,^40^ have also been employed for both Tb(iii) and Eu(iii) ions, or the acridone derivative for Eu(iii) (Fig. 2).^40^

Two main strategies have been used to prepare stable lanthanide metallopeptide complexes. The first approach involves using the peptide itself as a ligand for the metal ion, because peptide sequences can be easily adapted to coordinate Tb(iii) or Eu(iii) ions with high affinity.^29^ This is the case of short peptides based on the Ca(ii) binding loops of the calmodulin protein family, which can also coordinate Ln(iii) ions with micromolar affinity,^29,41^ or the optimized peptide sequences reported by Barbara Imperiali *et al.* based on the calcium-binding motifs of EF-hand proteins, also known as lanthanide binding tags (LBTs), which showed a Tb(iii) binding affinity in the low nanomolar range.^42^ These peptide sequences generally bind the lanthanide ions through multiple O and N donor atoms in the headgroups of carboxylic acids, such as aspartic acid (Asp) and glutamic acid (Glu) residues, or amides in asparagine (Asn) and glutamine (Gln), or in the peptide backbone. The incorporation of Trp residues as antenna in these lanthanide binding peptides resulted in luminescent complexes and transformed them into versatile genetically encoded tags for the study of proteins.^43–45^ The subsequent use of unnatural amino acids bearing polydentate ligands in their side chains, such as the tridentate aminodiacetate (Ada_2_) and the pentadentate ethylenediamine triacetate chelates (Ed3a_2_, Fig. 2), led to the development of peptide-based ligands with affinities in the femtomolar range for Tb(iii) ions.^46^

The second strategy for the preparation of stable lanthanide metallopeptides involves the modification of the peptide scaffold with a chelate known to form stable complexes with Ln(iii) ions, most commonly by the formation of an amide bond between the ligand and the peptide sequence. Most chelates used to coordinate Ln(iii) ions are polyaminocarboxylates, either linear, such as DTPA (diethylenetriaminepentaacetic acid, Fig. 2) or EDTA (ethylenediaminetetraacetic acid), or azacrown macrocycle derivatives, such as the cyclen (1,4,7,10-tetraazacyclododecane) derivatives DOTA (1,4,7,10-tetraazacyclododecane-1,4,7,10-tetraacetic acid) or DO3A (1,4,7,10-tetraazacyclododecane-1,4,7-triacetic acid) (Fig. 2). These macrocycles have a cavity of adequate size to accommodate lanthanide ions and multiple pendant carboxylate arms to form thermodynamically and kinetically stable complexes (Fig. 2).^47,48^

The simplest approach for their conjugation is to use commercial derivatives of acyclic ligands, such as EDTA or DTPA, including their bisanhydrides^49^ and their tris-/tetra-*tert*-butyl protected derivatives (EDTA(*t*Bu)_3_ and DTPA(*t*Bu)_4_),^50^ or macrocycles such as DOTA, as their NHS ester or tris-*t*Bu protected (DOTA(*t*Bu)_3_)^51–53^ derivatives. These are reacted with the *N*-terminal amino group, or even with orthogonally deprotected lysine (Lys) side chains or other amine-containing amino acids, of the peptide sequences bound to the solid support. Alternatively, tris-*t*Bu protected DO3A derivatives functionalized with a pendant amino group have also been successfully coupled to the carboxylic acids of orthogonally deprotected side chains of Asp and Glu residues.^54,55^ Given the number of bioconjugation reactions developed to date,^56–60^ functionalized chelates with isothiocyanate, maleimide, or alkyne moieties, such as DTPA or DOTA derivatives (and other polyaminocarboxylate-based macrocycles), are currently commercially available, making their incorporation into conveniently modified peptide sequences straightforward.

### Modulating the luminescence of lanthanide metallopeptides

The high coordination numbers of lanthanide ion complexes, together with the complex photophysics of the luminescence and sensitization process of Ln(iii) ions, allow a variety of strategies for the design of lanthanide metallopeptide-based biosensors.

**Fig. 3:**
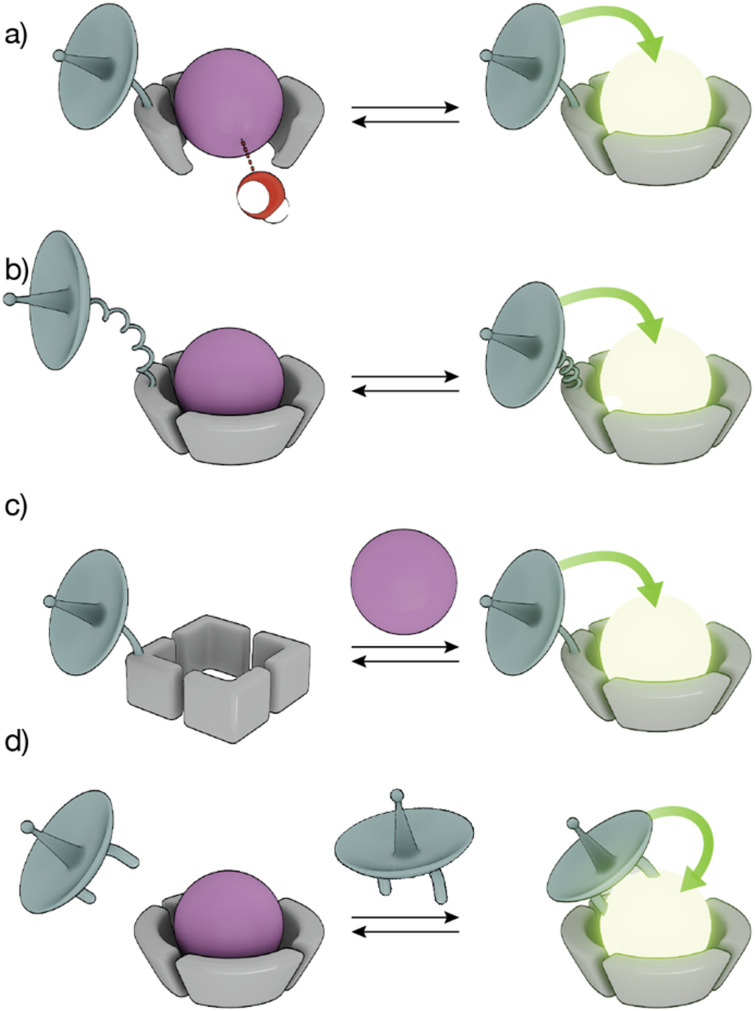
Strategies to modulate lanthanide sensitization and produce smart sensors. (a) The binding of an analyte displaces water molecules directly bound to the Ln(iii) ion, increasing the emission; (b) conformational changes reduce the distance between the sensitizer and the Ln(iii) ion, resulting in increased luminescence; (c) conformational or chemical changes in the peptide ligand increase the binding affinity for the Ln(iii) ion to generate the emissive Ln(iii)-metallopeptide probe; (d) direct binding of the Ln(iii) to the sensitizer yields an emissive complex.

The sensitivity of Ln(iii) ions, particularly Eu(iii) and Tb(iii) ions, to quenching by vibrational energy transfer to nearby N–H and O–H oscillators, such as the O–H oscillator of water, has been widely exploited to modulate the luminescence of metallopeptides by playing with the number of water molecules coordinated to the metal ion (Fig. 3a). In these cases, the lanthanide-binding ligand is designed to not satisfy the coordination of the metal ion so that its coordination sphere also includes water molecules. The interaction of the analyte with the Ln(iii) metallopeptide displaces the inner-sphere water molecule(s), thus increasing the emission intensity and the lifetime of the Ln(iii) ion complex.

Conformational changes that modify the distance between the antenna and the metal complex, and thus regulate the efficiency of the energy transfer, resulting in changes in the luminescence in the lanthanide metallopeptide complexes upon interaction with the analyte have also been employed to modulate the intensity of the Ln(iii) emission (Fig. 3b). Other sensing strategies exploit conformational or chemical changes in the peptide ligand induced by the analyte, to drastically modify its affinity for the Ln(iii) ion, so that only one state of the sensor coordinates the metal center resulting in a luminescent lanthanide metallopeptide complex (Fig. 3c).

A completely different approach has also been explored, using lanthanide metallopeptide complexes in which the antenna is not covalently attached to the metallopeptide. In some cases, the antenna directly coordinates to the Ln(iii) ion, resulting in a luminescent metallopeptide, but the presence or action of the analyte induces a change in the inner coordination sphere of the metal ion, thereby modifying the intensity of the emission of the Ln(iii). An alternatively strategy involves the supramolecular interaction of a non-luminescent lanthanide metallopeptide with an analyte that can effectively sensitize the Ln(iii), thus turning on the luminescence of the metal center (Fig. 3d). In the following sections, we will exemplify these different sensing mechanisms using representative biomolecule sensors based on lanthanide-metallopeptides.

## Luminescent lanthanide metallopeptides for biomolecule sensing and cellular imaging

The development of selective sensing systems for biomacromolecules, such as proteins or oligonucleotides, represents a major focus within the field of chemical biology, not only because of the interest in detecting these biomolecules, but also to understand their structural and functional properties. Ln(iii) metallopeptide platforms are promising biosensing agents, given the aforementioned advantages of these metal ions and the potential for recognizing specific targets through their conjugation to peptide fragments. Thus, several Ln(iii) metallopeptides have been developed as reporters for the study of specific molecules, including peptide/proteins^44,45,51,52,61^ and nucleic acids,^49,53,62,63^ as well as to monitor of enzyme activity^64–66^ and post-translational modifications (PTMs), either generated enzymatically, *e.g.* phosphorylation/dephosphorylation^54,67–74^ or in response to oxidative or nitrosative stress.^55,75–77^

### Protein and peptide sensing

**Fig. 4:**
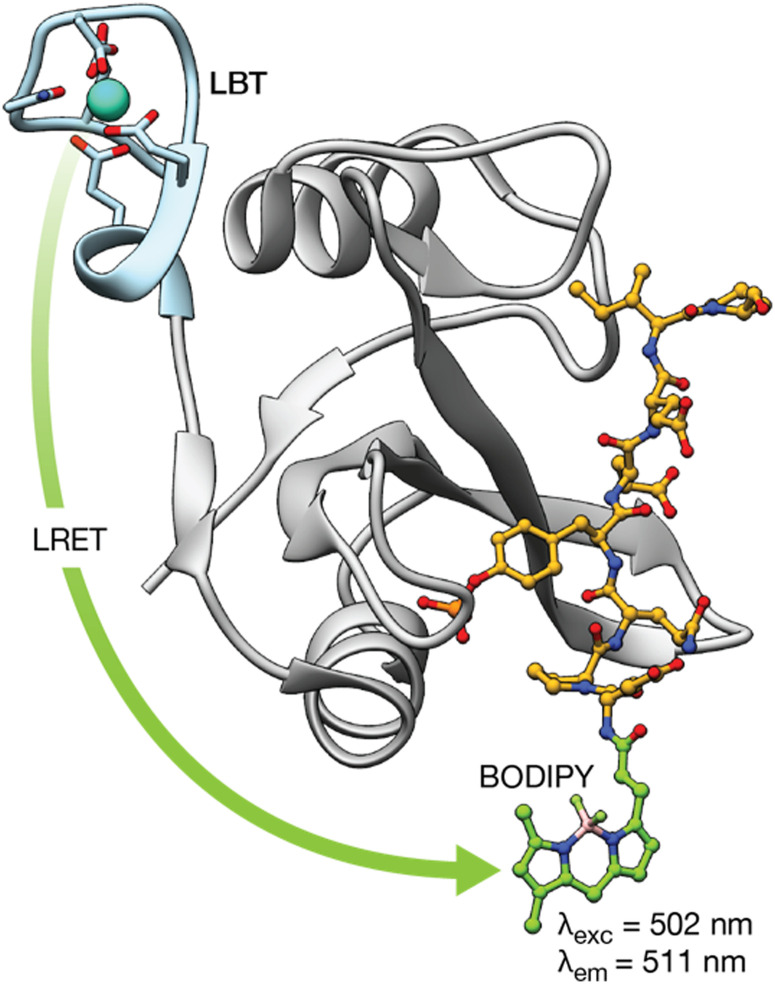
LRET transfer from the LBT–Tb(iii) tag attached to the Src-SH2 domain to the *N*-terminal BODIY phosphopeptide used to detect the interaction of the Src-SH2 domain with different phosphopeptides. In addition, Förster theory can be used to calculate the distance between the lanthanide ion and a site in the bound phosphopeptide using decay experiments. Adapted with permission from ref. 44. Copyright 2006 American Chemical Society.

Proteins regulate a multitude of cellular processes, playing a fundamental role in cell growth, division, metabolism, or signaling, among others. Therefore, understanding protein structure, function, and dynamics is crucial in biology, chemical biology, and biochemistry. This has led to the development of lanthanide-binding metallopeptides as effective biosensors for studying proteins and monitoring their interactions with other proteins.^44^ A seminal work in this area was reported by the group of B. Imperiali in 2006, who established a novel methodology demonstrating that LBTs can be fused to proteins of interest and used to monitor protein–protein interactions through luminescence resonance energy transfer (LRET).^44^ LRET is a method in which a previously sensitized Ln(iii) ion functions as a donor and transfers its energy to an acceptor fluorophore. Therefore, in this work, the group expressed the SH2 domains of Src and Crk kinases attached to LBT tags, and used them to monitor the interaction with peptides and phosphopeptides modified with organic fluorophores at their *N*-termini. This method allowed the measurement of the binding affinities of phosphorylated and non-phosphorylated peptides for the SH2 domains. Moreover, by performing decay experiments and using Förster theory, it was possible to calculate the distances between the Tb(iii) LBT complex and the organic BODIPY fluorophore in the peptides upon formation of the SH2/peptide complexes (Fig. 4).

Subsequent works have employed this LRET strategy to study protein interactions between oligosaccharyl transferases and fluorescently labeled peptide and glycan substrates,^45^ as well as molecular chaperones such as HSP70 and HSP90 with their co-chaperone HOP labeled with GFP.^61^

**Fig. 5:**
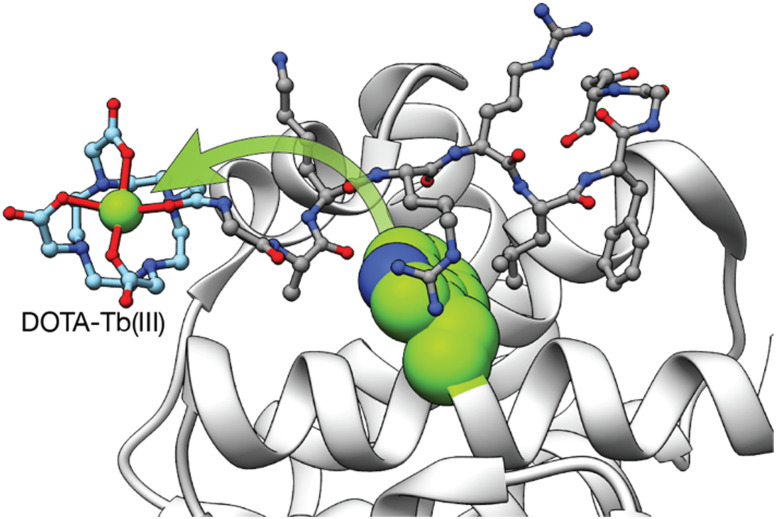
Intermolecular Tb(iii) sensitization from the Trp in the vicinity of the cyclin A binding grove used to detect the interaction between the Tb(iii) metallopeptide and cyclin A. Adapted with permission from ref. 51 Copyright 2008 American Chemical Society.

In addition to the use of genetically encoded LBTs for the study of protein interactions, we reported two different synthetic Tb(iii) metallopeptides for the detection of proteins involved in cancer.^51,52^ In 2008, we incorporated a DOTA–Tb(iii) at the *N*-terminus of a short peptide that mediates specific cyclin A binding. The absence of an antenna on the metallopeptide resulted in a non-luminescent probe. However, upon binding to cyclin A, the DOTA–Tb(iii) complex is placed in close proximity to a Trp residue of the cyclin A binding groove so that the intermolecular sensitization of the Tb(iii) switches on its luminescence (Fig. 5).^51^ Although this example demonstrated the possibility of sensing proteins by means of intermolecular sensitization of Tb(iii) metallopeptides, its applicability to the sensing of other biomolecules is limited due to the need of an adequate antenna near the binding site of the probe for an efficient energy transfer process to occur.

Three years later, we reported a novel strategy for detecting coiled coil proteins based on the modulation of the energy transfer between the antenna and the Tb(iii) complex acceptor, in response to the folding of the peptide chain upon coiled coil dimer formation.^52^ The designed metallopeptide probe ^W17^K_4_ features a DOTA–Tb(iii) complex, bound to a 2,3-diaminopropanoic acid side chain, and a Trp residue at a position *i* + 4 (so their distance is minimized upon folding into an α-helical conformation). Since coiled coil peptide monomers are unfolded in their monomeric state but fold into α-helical structures when forming the coiled coil dimer, ^W17^K_4_ exhibits weak luminescence emission in solution. However, the presence of the complementary E_4_ peptide triggers the folding of the peptides, thus inducing an increase in the luminescence emission intensity. Importantly, we demonstrated that this methodology can be translated to the detection of the oncogenic transcription factor c-Jun, which belongs to the leucine zipper family of transcription factors and adopts a coiled coil structure when it dimerizes with c-Fos. The corresponding metallopeptide probe A-Fos^W^[Tb] incorporates the Trp and DOTA–Tb(iii) complex in equivalent positions to that of ^W17^K_4_. In agreement with the model system, A-Fos^W^[Tb] showed an increase in luminescence intensity upon incubation with the DNA-binding domain of c-Jun. Overall, this approach set the basis for the study of biological interactions involving the folding of disordered proteins into well-defined secondary structures.

### Nucleic acids sensing

Although lanthanide complexes have gained significant attention in the field of DNA research with numerous examples employing lanthanide complexes for DNA sensing,^78,79^ most of these examples are lanthanide complexes that bind DNA with poor selectivity through DNA intercalation or involve the terminal modification of nucleic acid sequences with lanthanide complexes and organic chromophores for the detection of the target ssDNA through hybridization assays. In this context, the use of lanthanide-based metallopeptides for DNA sensing appears to be rather limited and, to the best of our knowledge, only the group led by Delangle has used peptides for this purpose.^62^ In this work, the authors described an Eu(iii)-binding peptide ligand with two unnatural Ada_2_ amino acids, which coordinate the europium ion with nM affinity, and a DNA-intercalating proflavine (Pfl) moiety. The time-resolved emission spectra of the resulting EuPfl-P^22^ complex showed the characteristic Eu(iii) emission bands upon excitation of the Pfl unit at 450 nm, which serves as the sensitizer. In the presence of calf thymus DNA, the lanthanide emission is quenched by the intercalation of the aromatic proflavine into double-stranded DNA. Although the authors demonstrated a luminescent response after DNA binding, the DNA affinity constant of the probe is in the same range as the proflavine unit itself and lacks DNA selectivity, thus limiting its practical application.

In terms of nucleic acid recognition, RNA has been increasingly recognized as a fundamental element in the biochemistry of cells,^80^ playing a critical role in gene expression or in post-transcriptional events through non-coding RNAs, with crucial functions in multiple disease states.^81–83^ Therefore, the development of new diagnostic tools for these biomarkers has also gained attention. Building upon previous work on sensing coiled coil proteins, we described two different designs of luminescent lanthanide metallopeptide probes capable of detecting secondary RNA structures with high selectivity, based on binding-induced conformational changes in the probes.^49,53^ In 2013, a short peptide sequence, based on the RNA binding domain of the transcriptional antitermination protein N of phage P22 (P22-N), was functionalized with a DOTA–Tb(iii) complex attached to a Lys side chain and a Trp residue located four residues away from the metal complex (*i, i* + 4 separation).^53^ The interaction of this P22-N^W^[Tb] metallopeptide with the short RNA hairpin boxB target triggers the folding of the peptide from a random coil conformation into an α-helix, resulting in an increase of its luminescence intensity due to the reduced distance between the emissive DOTA–Tb(iii) complex and the Trp antenna. Importantly, the probe showed good affinity for boxB (in the low nanomolar range) and selectivity over single- and double-stranded DNA oligonucleotides and the related bovine immunodeficiency virus TAR hairpin.

In 2016, we reported a lanthanide metallopeptide probe targeting the bovine immunodeficiency virus (BIV) RNA transactivation response element (TAR), which is related to the human immunodeficiency virus (HIV).^49^ In this case, the sensing mechanism is based on the large conformational change of the EDTA[Ln]-Tat-Lys(ϕ) metallopeptide upon binding to its target, from an extended random coil structure to a compact β-hairpin. This folding places a phenanthroline coordinating antenna (ϕ) nearby the EDTA[Ln] complex (attached to the *C*- and *N*-terminus, respectively), displacing a water molecule from the metal complex and resulting in a 12-fold increase in the luminescence intensity of the Tb(iii) complex and a 30-fold increase in the case of the Eu(iii) analog. The EDTA[Ln]-Tat-Lys(ϕ) metallopeptide binds TAR with high affinity and selectivity, with a detection limit in the low nanomolar range, an RNA concentration below that of infected cells. Furthermore, we demonstrated the possibility of performing multiplex sensing by combining EDTA[Eu]-Tat-Lys(ϕ) with P22-N^W^[Tb], showing that both probes are capable of independently signaling their respective target hairpin RNAs, TAR and boxB.

**Fig. 6:**
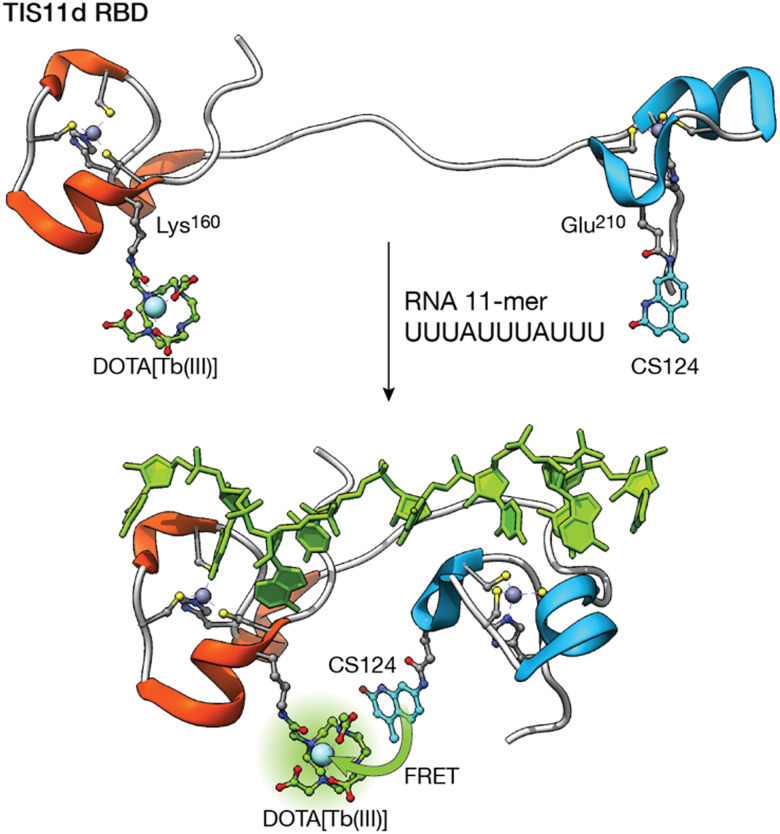
Folding-upon-binding as the mechanism for the detection of AU-rich elements. The DOTA–Tb(iii) complex and the carbostyril 124 sensitizer are located in two zinc finger domains separated by a flexible linker. RNA recognition brings them together, maximizing the sensitization upon binding. Adapted from ref. 63 with permission from the Royal Society of Chemistry.

Following a similar strategy, the group of O. Sénèque described a Tb(iii) metalloprotein-based sensor that selectively detects short RNA sequences located in the adenylate–uridylate-rich elements (AU-rich elements) of the 3′-untranslated region of mRNA. The sensor is based on the tristetraprolin (TTP) protein family, which binds this RNA as part of their role in the regulation of inflammation and cancer.^63^ More specifically, the designed LTIS^Tb^ metalloprotein is a 68 amino acid sequence, based on the RNA binding domain of the TIS11d protein of the TTP family, consisting of two zinc finger domains separated by a 10-residue flexible linker. One of the zinc finger domains is functionalized with a DOTA–Tb(iii) complex and the other with carbostyril 124, so that recognition of the AU-rich RNA sequence leads to an increase in the luminescence emission from the Tb(iii) ion as a consequence of the conformational change of the probe upon RNA binding (Fig. 6). Interestingly, the authors showed that it is possible to synthesize long metalloprotein-based probes by assembling three unprotected and conveniently modified peptide fragments in water by combining native chemical ligation and SEA ligation, thus demonstrating that the development of biomolecule probes is not limited to those based on short peptide fragments.

### Monitoring enzyme activity and non-enzymatic protein modifications

Lanthanide complexes have been widely explored to monitor enzymatic activity, including that of glycosidases, lipases, and esterases, among others.^27,84^ However, the application of lanthanide metallopeptides to probe enzyme activity has been rather limited, and most of the studies are focused on monitoring of kinases and phosphatases,^20^ and, to a lesser extent, proteases.

Proteases play a role in many biological pathways, including cell signaling and apoptosis. Additionally, some microorganisms secrete proteases as virulence factors, thereby increasing their pathogenicity. However, despite their biological relevance, there are very few lanthanide metallopeptides for monitoring the activity of proteases, all of them based on two related sensing strategies. In 2004, the group of Karvinen described the use of lanthanide metallopeptides for monitoring the activity of caspases,^64,65^ using a caspase-specific substrate modified at the *C*-terminus with a Lys residue and at the *N*-terminus with a Cys residue; these two residues were covalently modified with an isothiocyanate-activated QSY7 dye and an iodoacetamide-chelate. In this case, the QSY7 dye works as a non-fluorescent acceptor (quencher) of the luminescent lanthanide complexes. When present, the protease cleaves the peptide, separating the Ln(iii) complex and the quencher, thereby restoring the luminescence of the metal center.^64^ By combining different lanthanide complexes (Tb(iii), Eu(iii), and Sm(iii)) and peptide substrates for different caspases, the authors demonstrated the possibility of multiplexing the activity of caspase-1, 3, and 6.^65^ Approximately ten years later, the group of Vuojola showed that LRET can also be used to monitor protease activity.^66^ The designed probe consisted of the LBT–Tb(iii) complex, previously reported by the Imperiali group,^44^ acting as the luminescence donor and a green fluorescent protein (GFP) as the acceptor, both connected by a caspase-3 recognition sequence, so that LRET occurs between the two. However, caspase-3 cleaves the peptide linker, thus deactivating the fluorescence of GFP due to the separation of the LBT–Tb(iii) complex and the GFP. Importantly, the authors demonstrated the applicability of the assay for caspase-3 inhibitor screening. We have recently reported short luminescent Tb(iii) and Eu(iii)-metallopeptide sensors for monitoring the activity of elastase B, the main extracellular virulence factor of *Pseudomonas aeruginosa*.^85^ The probes resisted degradation by other proteases and remained stable in the presence of bioanalytes related to *P. aeruginosa* infections and in complex biological media. Importantly, time-gated experiments removed the characteristic background fluorescence of *P. aeruginosa* cultures, showing its potential for real-time monitoring of the protease in bacterial cultures.

Post-translational modifications (PTMs) are covalent transformations of amino acid sidechains (*e.g.*, Lys acetylation, Ser, Thr, Tyr, phosphorylation, Asn, Ser and Thr glycosylation, or S-nitrosylation of Cys) responsible for controlling the structure and function of proteins and, as such, represent a distinct category among the numerous cell regulatory mechanisms. Protein phosphorylation is a reversible enzyme-driven PTM that is involved in a myriad of fundamental cellular processes and is strongly associated with various diseases, including cancer.^86^ In this context, luminescent probes provide a powerful tool not only to study the biochemical function and regulation of the reversible protein phosphorylation mechanism, but also to identify possible inhibitors of the enzymes. Many examples of lanthanide-based metallopeptides for the study of kinases and phosphatases have been reported and previously reviewed.^20,27,84^ Therefore, this section will only present a few representative examples of the different strategies used in their design.

**Fig. 7:**
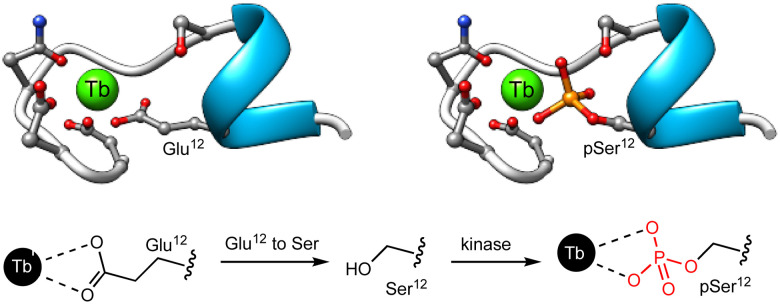
Representation of the EF hand motif, in which the Glu residue has been replaced by a Ser residue that has poor affinity for Tb(iii). Phosphorylation by PKA yields the corresponding phosphoserine, forming a high affinity complex with Tb(iii). Adapted with permission from ref. 67. Copyright 2006 American Chemical Society.

Given that the phosphorylation state of a peptide can have a drastic effect on its affinity for Ln(iii) ions,^87,88^ the first examples of Ln(iii) metallopeptide sensors of PTMs leveraged this PTM to control the metal coordination as a way to modulate the luminescence of lanthanide metallopeptides. In 2006, the group of Neal J. Zondlo modified the sequence of an EF hand motif by substituting a Glu residue that is fundamental for Tb(iii) binding with a Ser residue, and included at its *C*-terminus a minimal consensus sequence for several kinases, such as mitogen-activated protein kinase (MAPK), protein kinase A (PKA), and protein kinase C (PKC).^67^ The designed peptides were non-luminescent due to the poor binding to Ln(iii) ions of the sequences featuring the Ser residue, whereas their phosphorylated analogues formed 1 : 1 complexes with Tb(iii) and exhibited strong luminescence from the metal center (Fig. 7). The authors showed that incubation with PKA increases the Tb(iii) luminescence as it forms a high-affinity complex with the phosphopeptide featuring a phosphorylated serine residue (pSer), and demonstrated that this approach can be adapted to monitor other kinases, such as Abl,^68^ PKC, casein kinase 1 (CK1), and even calf intestine alkaline phosphatase (CIP).^69^ In this last work, they developed a minimal peptide motif of 7–8 amino acids combined with a Ser or Thr at position 9, which can be phosphorylated to generate high-affinity Ln(iii) metallopeptides.

Related works have used phosphotyrosine (pTyr) peptides, in which the phosphorylated residue serves not only to enhance the affinity for the lanthanide ion but also as a sensitizer, to monitor the activity of ALK,^70^ Abl, Jak2, Src,^71^ and BTK^72^ tyrosine kinases.

**Fig. 8:**
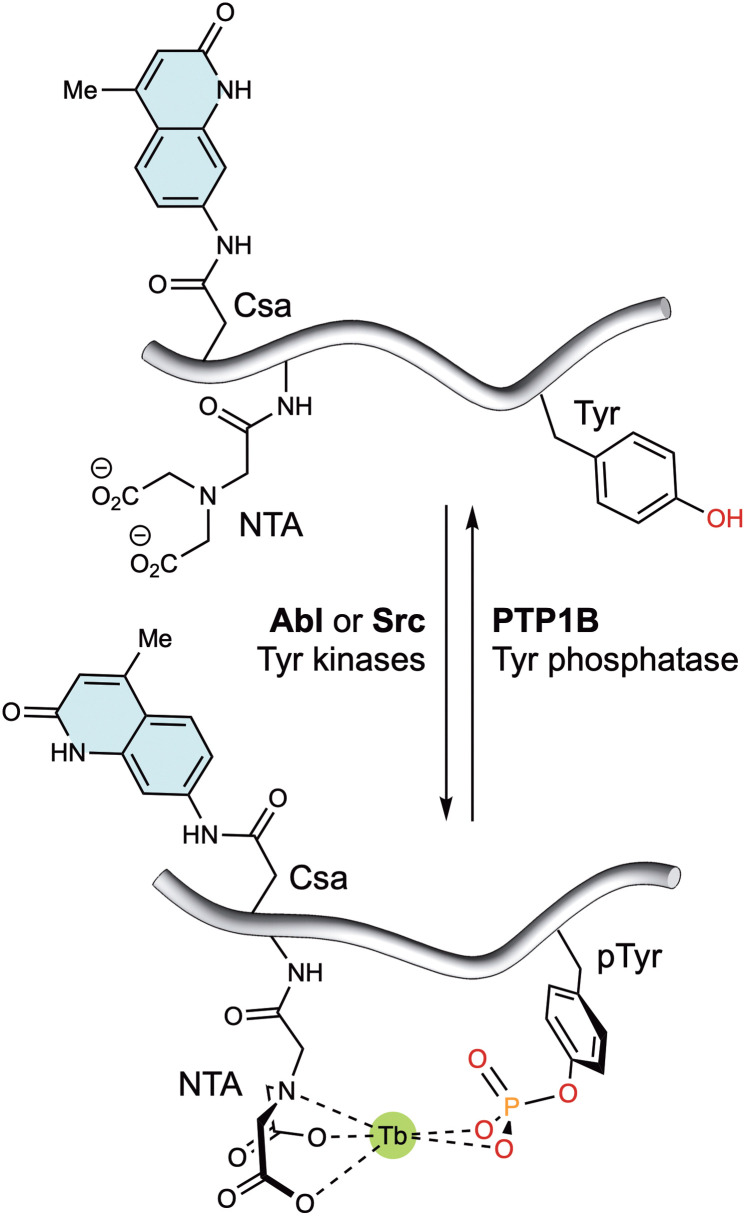
Tyrosine kinase/phosphatase sensor based on the generation of a suitable coordination environment to bind Tb(iii) upon phosphorylation by Abl and Src kinases. The carbostyril 124 chromophore in Csa can sensitize both Tb(iii) and Eu(iii), allowing the long-wavelength readout of this system. Dephosphorylation with PTP1B leads to decomplexation of the lanthanide ions and a marked reduction in the luminescent emission. Adapted with permission from ref. 73. Copyright 2008 American Chemical Society.

Alternatively, lanthanide metallopeptides containing non-natural chelating residues and antennae have also been designed to monitor enzyme activity. Thus, the group of Dalibor Sames reported a short peptide probe containing an imidodiacetate ligand and a carbostyril 124 antenna near the phosphorylation site. This probe exhibits enhanced luminescence upon tyrosine phosphorylation, due to the higher affinity of the probe for both Eu(iii) and Tb(iii) ions (Fig. 8). Notably, the probe responds to its phosphorylation by Abl and Src kinases, as well as dephosphorylation by PTP1B phosphatase.^73^

Following a different strategy, we developed a lanthanide metallopeptide for the luminescent detection of reversible phosphorylation events.^54^ The designed peptide contains a *C*-terminal PKCα recognition sequence and a DO3A–Eu(iii) complex covalently attached to an Asp side chain, separated from the phosphorylation site (Ser) by a Pro-Gly β-turn. Incubation of the unphosphorylated and coordinatively unsaturated Eu(iii) metallopeptide 1[Eu] with the external antenna trifluoro-1-(2-naphthyl)-1,3-butanedione (TNB) leads to the formation of the 1[Eu]·TNB metallopeptide complex, in which the TNB antenna is directly coordinated to the metal ion, filling all the coordination positions of the ion, and thus producing a high intensity emission. Phosphorylation of the Ser results in the intramolecular coordination of the phosphate group to the metal ion and displacement of the antenna, thus inactivating the luminescence emission from the Eu(iii) ion. Interestingly, both the phosphorylated 1^P^[Eu] and unphosphorylated 1[Eu]·TNB metallopeptides allowed the real-time monitoring of alkaline phosphatase and PKCα, respectively.

The group of Morris later described a Tb(iii) metallopeptide to monitor CDK4 kinase activity in melanoma cell extracts, using a different sensing mechanism.^74^ The designed metallopeptide consists of two regions: (i) a CDK4 substrate sequence incorporating a DOTA–Tb(iii) complex covalently attached to a Cys side chain, located two residues away from the phosphorylation site, and (ii) a phosphoamino acid binding domain (PAABD) form the interface of the WW domain of Pin1, which includes the Trp antenna. When the metallopeptide is unphosphorylated, the intramolecular energy transfer between the antenna and the DOTA–Tb(iii) complex is less efficient because of the large distance between the two units. In turn, phosphorylation of the Ser residue promotes a conformational change in the metallopeptide facilitated by the recognition of the pSer residue by the PAABD region. This results in the relocation of the Trp residue closer to the metal ion, thereby enhancing the efficiency of the energy transfer and the luminescence of the metallopeptide.

In addition to enzymatically driven PTMs, proteins may undergo a series of non-enzymatic PTMs because of oxidative or nitrosative stress, including cysteine S-nitrosylation, sulfinic acid formation, or glutathionylation, as well as tyrosine nitration, among others. While some allow protein responsiveness to changes in the redox state of the cell, others, such as tyrosine nitration, are considered pathological markers and can induce different effects on protein function. Consequently, there is an increasing interest in the development of luminescent probes for the specific detection of PTMs. In this regard, lanthanide metallopeptides are particularly attractive because they can reproduce the natural substrates involved in these PTMs. The group of Neal J. Zondlo has reported several lanthanide metallopeptides for monitoring PTMs, such as cysteine glutathionylation,^75^ cysteine S-oxidation to sulfinic acid,^76^ and tyrosine nitration.^77^ Similar to their approach for monitoring protein phosphorylation,^67^ substitution of carboxylate residues of the EF hand motif involved in Tb(iii) binding, such as Asp-5 or Glu-12, with a Cys residue resulted in peptide sequences with modest Tb(iii) affinity. However, the Cys glutathionylation^75^ or sulfinic acid oxidation^76^ increased the affinity of the peptides for the Tb(iii) ion, and thus their luminescence intensity, due to the coordination of the free carboxylate of the conjugated glutathione or sulfinate side chain, respectively, to the Tb(iii) ion. Conversely, the substitution of the Glu-12 residue of the EF hand motif with a Tyr residue resulted in a peptide with moderate affinity for Tb(iii), whereas nitration of this residue led to a significantly increase in affinity for the metal ion, accompanied by an approximately three-fold reduction in luminescence.^77^ The developed EF hand domain was encoded as a tag at the *C*-terminus of maltose-binding protein, exhibiting luminescence that was quenched in response to the amount of peroxynitrite added. In this context, we have recently developed a short synthetic metallopeptide for monitoring nitration states.^55^ The peptide is based on the sequence of α-synuclein, functionalized with a DO3A–Tb(iii) complex, and exhibits a large change in luminescence intensity (approximately 40-fold) between the non-nitrated and nitrated states of the tyrosine residue in the metallopeptide. It is noteworthy that the decrease in luminescence intensity observed when the probe is incubated with different concentrations of peroxynitrite, correlates with the degree of tyrosine nitration. Furthermore, the response of the probe remains unaffected in complex biological media.

### Cell imaging with lanthanide metallopeptides

Although many of the described lanthanide metallopeptides work well in complex biological media, such as in cell lysates, a major drawback of the previous examples is that they use sensitizers that are excited in the 260–300 nm region. This limits their application for *in vitro* sensing and imaging due to the characteristics of microscope objectives that do not transmit light below 350 nm, and the high absorbance of biological samples in the blue region of the spectrum. Moreover, despite their many advantages, the low photon efflux of lanthanide probes have limited their application in fluorescence microscopy.^89^ These drawbacks have been overcome by some synthetic lanthanide metallopeptides containing antennas that can be excited above 350 nm, or by two-photon excitation in the near infrared range.^90–94^ In 2003, the group of Thomas J. Meade reported a lanthanide metallopeptide comprising a DOTA–Eu(iii) complex conjugated to the *N*-terminus of an Arg_16_ peptide, which has no chromophores in its structure. Due to the polyarginine tail, the metallopeptide was internalized by NIH 3T3 cells and imaged for the first time by direct excitation of the Eu(iii) ion at 750 nm by two-photon laser microscopy.^90^ Later, Ka-Leung Wong and coworkers were able to image cyclin A in HeLa cells with an Eu(iii) metallopeptide. In this case, the authors modified the *N*-terminus of a peptide sequence, known to bind specifically to the cyclin A binding groove, with a DO3A–Eu(iii) complex directly conjugated to an aryl–alkynyl–pyridine antenna. They showed that the metallopeptide is internalized by HeLa cells, as observed by ICP–MS and by two-photon microscopy after excitation at 800 nm. Notably, the intensity of the luminescence was reduced when cyclin A expression was inhibited, indicating the binding of the metallopeptide to the target protein within the cells.^91^ In a recent work, O. Sénèque and colleagues described the cytosolic delivery of Eu(iii) and Tb(iii) metallopeptides by two-photon microscopy. The metallopeptides consist of a disulfide dimer of the cell-penetrating peptide transactivator of transcription of human immunodeficiency virus (TAT), which is modified in a Lys side chain with several DO3A–aryl–picolinate derivatives. Both Eu(iii) and Tb(iii) metallopeptides were internalized by HeLa, HEK293T and MRC5 cells, and delivered to the cytosol as evidenced by the diffuse Tb(iii) or Eu(iii) emission observed within the entire cell imaged after excitation at 720 nm. The authors confirmed that the dimerization of the TAT peptide is essential for the cytosolic delivery of the metallopeptides. However, although the nature of the aryl–picolinate antennas influences the cytotoxicity of the metallopeptides, it has no impact on their cellular localization.^92^ Curiously, a previous work from the same group showed that the nature of the lanthanide complex can influence the internalization properties of the cell-penetrating peptides used as internalization vectors.^93^ In this case, none of the metallopeptides containing a DO3A–aryl–picolinate–Tb(iii) complex or a DO3A–aryl–alkynyl–picolinate–Eu(iii) complex attached to the TP2 cell-penetrating peptide were internalized by NIH3T3 cells, since no luminescence was detected by time-gated microscopy after excitation of the antenna at 337 nm. However, when the same DO3A–aryl–picolinate–Tb(iii) complex is attached to the ZF5.3 cell-penetrating peptide, which promotes early-endosome escape, the metallopeptide is internalized by NIH3T3 and HeLa cells. In both cases, the cytosolic distribution of the metallopeptide was imaged by one-photon time-gated photoluminescence microscopy (NIH3T3 cells, *λ*_ex_ = 337 nm), removing the background autofluorescence of the sample, and two-photon confocal microscopy (HeLa cells, *λ*_ex_ = 700 nm). Similarly, the group of David Parker reported the effect of the linker connecting a triazacyclononane–Eu(iii) complex to the Cys side chain of a peptide sequence targeting the endoplasmic reticulum. The authors showed that direct conjugation of the Cys side chain to the pyridyl arm of the triazacyclononane has no impact on the targeting of the resulting Eu(iii) metallopeptide, which was observed to be localized in the endoplasmic reticulum of NIH 3T3 cells by confocal microscopy (*λ*_ex_ = 355 nm). Conversely, the conjugation of the Cys to a pendant maleimide–amino–propyl linker attached to the same pyridyl arm of the macrocycle changes the localization of the resulting Eu(iii) metallopeptide, which was found in the lysosomes by confocal microscopy.^94^

Although less common than Tb(iii) or Eu(iii) metallopeptides, K.-L. Wong and coworkers have described other near-infrared (NIR) emitting lanthanide metallopeptides.^95–97^ By combining different targeting peptide sequences, lanthanide complexes, and antennas, they developed a number of imaging or theragnostic agents. In their earlier examples, the conjugation of an Er(iii)–porphyrin complex to a short peptide sequence that binds the integrin α_v_β_3_ isoform resulted in a metallopeptide with high selectivity for blader cancer cells. In addition to the strong NIR emission (*λ*_ex_ = 430 nm) that allows localization of the metallopeptide in the lysosomes of blader cancer cells, the incorporation of the porphyrin unit provides a multimodal agent for its use in photodynamic therapy *via*^1^O_2_ generation that kills cancer cells.^95^ Later, the incorporation of a DO3A–aryl–alkynyl–pyridine–Yb(iii) complex at the *N*-terminus of peptide P19 through a copper(i)-catalyzed alkyne–azide cyclo-addition afforded the corresponding YbP19 metallopeptide, which was designed to bind latent membrane protein 1 (LMP1). This is the major transforming protein of the Epstein–Barr virus (EBV) and is implicated in a variety of EBV-associated cancers. The YbP19 metallopeptide allowed live NIR imaging of LCL3 cancer cells (*λ*_ex_ = 370 nm), while it showed no NIR signal when incubated with LMP1-negative HeLa cells. In addition, the probe exhibited high cytotoxicity against LMP1-expressing cells by suppressing the NF-*κ*B pathway, thus providing an alternative treatment for cancers with positive LMP1 expression.^96^ In a recent publication, these same authors have developed a straightforward methodology for the synthesis of dual-emitting Nd(iii) metallopeptides. By incorporating a Lys residue at the *N*-terminus of a target peptide sequence to which a 7-nitrobenzofurazan (NBD) chromophore and a DO3A derivative are attached in the side chain and terminal amino group, respectively, while the peptide is bound to the solid support. Several Nd(iii) metallopeptides were prepared through this simple approach. The DO3A–Nd(iii) complex and the NBD antenna pair were successfully incorporated into a mitochondria localization sequence (MLS) and an Epstein–Barr virus nuclear antigen 1 (EBNA1) targeting peptide, with the corresponding Nd(iii) metallopeptides exhibiting dual emission in the visible (*λ*_em_ ≈ 555 nm) and NIR (*λ*_em_ ≈ 1059 nm) regions after excitation at ≈ 480 nm, enabling their localization in the mitochondria of HeLa cells and the nucleus of EBNA1-positive cell lines, respectively.^97^

## Outlook and future perspectives

The unique photophysical properties of lanthanide metallopeptides, such as their long lifetimes and relatively narrow emission bands, combined with the targeting properties and responsiveness to cellular events of peptide sequences, allow a variety of sensing strategies for the design of smart biosensors and imaging agents. Consequently, lanthanide metallopeptides have been exploited to develop luminescent probes for the detection of peptides, proteins and nucleic acids, as well as for monitoring enzymatic activity and PTMs. Although the study of protein phosphorylation/dephosphorylation events has been the primary focus of luminescent lanthanide metallopeptide probes, future work will likely expand to other relevant biomarkers and explore alternative sensing mechanisms.

Lanthanide metallopeptides have also been used as imaging agents, although in these examples the peptide moieties serve merely as internalization vectors that direct the lanthanide complexes to specific organelles/targets. On the other hand, genetically encoded biosensors have been employed to monitor protein interactions in cells by LRET.^24,98,99^ Nevertheless, these require cell transfection and over-expression, and when aiming for *in vivo* sensing, these may exhibit poor biodistribution due to their large size, thus limiting their applicability. Therefore, future developments will likely evolve in this direction, providing smart lanthanide metallopeptide probes that allow for the direct visualization of dynamic changes in the location, interactions, or activity of biomolecules within living cells at longer wavelengths.

## Author contributions

Conceptualization: E. P. and M. E. V.; writing – original draft: R. S. F., I. O. G., A. S., M. E. V., and E. P.; writing – review and editing: M. E. V. and E. P.; supervision: E. P.; funding acquisition: M. E. V. and E. P. All authors read and approved the final manuscript.

## Data availability

No primary research results, software or code have been included and no new data were generated or analyzed as part of this review.

## Conflicts of interest

There are no conflicts to declare.
